# Key Players in the Mutant p53 Team: Small Molecules, Gene Editing, Immunotherapy

**DOI:** 10.3389/fonc.2020.01460

**Published:** 2020-08-18

**Authors:** Vitaly Chasov, Regina Mirgayazova, Ekaterina Zmievskaya, Raniya Khadiullina, Aygul Valiullina, Joseph Stephenson Clarke, Albert Rizvanov, Matthias G. J. Baud, Emil Bulatov

**Affiliations:** ^1^Institute of Fundamental Medicine and Biology, Kazan Federal University, Kazan, Russia; ^2^School of Chemistry, University of Southampton, Southampton, United Kingdom; ^3^Shemyakin-Ovchinnikov Institute of Bioorganic Chemistry, Russian Academy of Sciences, Moscow, Russia

**Keywords:** p53, mutation, small molecules, adenoviral gene therapy, CRISPR/Cas gene editing, immunotherapy

## Abstract

The transcription factor p53 is a key tumor suppressor that is inactivated in almost all cancers due to either point mutations in the *TP53* gene or overexpression of its negative regulators. The p53 protein is known as the “cellular gatekeeper” for its roles in facilitating DNA repair, cell cycle arrest or apoptosis upon DNA damage. Most p53 mutations are missense and result in either structural destabilization of the protein, causing its partial unfolding and deactivation under physiological conditions, or impairment of its DNA-binding properties. Tumor cells with p53 mutations are generally more immunogenic due to “hot spot” neoantigens that instigate the immune system response. In this review, we discuss the key therapeutic strategies targeting mutant p53 tumors, including classical approaches based on small molecule intervention and emerging technologies such as gene editing and T cell immunotherapy.

## Introduction

The transcription factor p53 functions as a tumor suppressor and is considered as one of the most promising molecular targets for cancer therapy, as it regulates a plethora of intracellular metabolic pathways, including DNA damage repair, apoptosis, and senescence. The p53 protein is widely known as the “guardian of the genome” that prevents the proliferation of cells harboring genetic aberrations, notably oncogenic mutations. In both stressed and unstressed cells, the p53 protein is subject to post-translational modifications, including phosphorylation, acetylation, ubiquitination, and methylation that regulate its stability, localization (cytoplasm or nucleus) and transcriptional activity. Phosphorylation of Ser or Thr residues of p53 was shown to correlate with increasing of p53 activity in response to cellular stress (1).

The *TP53* gene encoding the p53 protein is the most frequently altered gene in human tumors (2). The loss of transcriptional functions leading to the deactivation of intrinsic tumor suppressive responses associated with wild-type (WT) p53 is the primary outcome of p53 mutations, and is a hallmark of most cancers The majority of p53 mutations are missense, i.e., cause single residue substitutions, and occur within the DNA-binding domain (DBD). These can be classified as either “DNA contact” or “conformational” mutations (3). “DNA contact” mutations occur in regions that make direct contact with target DNA sequences and are critical for DNA binding, whereas “conformational” mutations diminish DNA-binding by distorting the protein structure through destabilization. Most of these mutations are loss-of-function and exert a dominant negative effect on the WT protein functions. Beyond this, cancer cells appear to gain selective advantages by retaining only the mutant form of the protein, associated with enhanced cell proliferation, metastasis and chemoresistance (4).

The intracellular p53 level is tightly regulated by its negative regulator murine double minute 2 (MDM2) ubiquitin ligase, mostly through ubiquitination followed by proteasomal degradation. In most human cancers, p53 is deactivated either due to mutation or because of the overexpression of MDM2. The strategy of enhancing p53 functions by means of small molecule MDM2 inhibitors has long been of interest to the field by its perceived tractability (5). However, despite development of dozens of high-affinity compounds and multiple clinical trials, none have yet produced a registered drug, suggesting that alternative paths should be given greater attention (6). The MDM2-induced degradation of p53 could be regulated by p14ARF that inhibits the oncogenic action of MDM2 and enhances p53-dependent transactivation and apoptosis (7).

**Figure 1:**
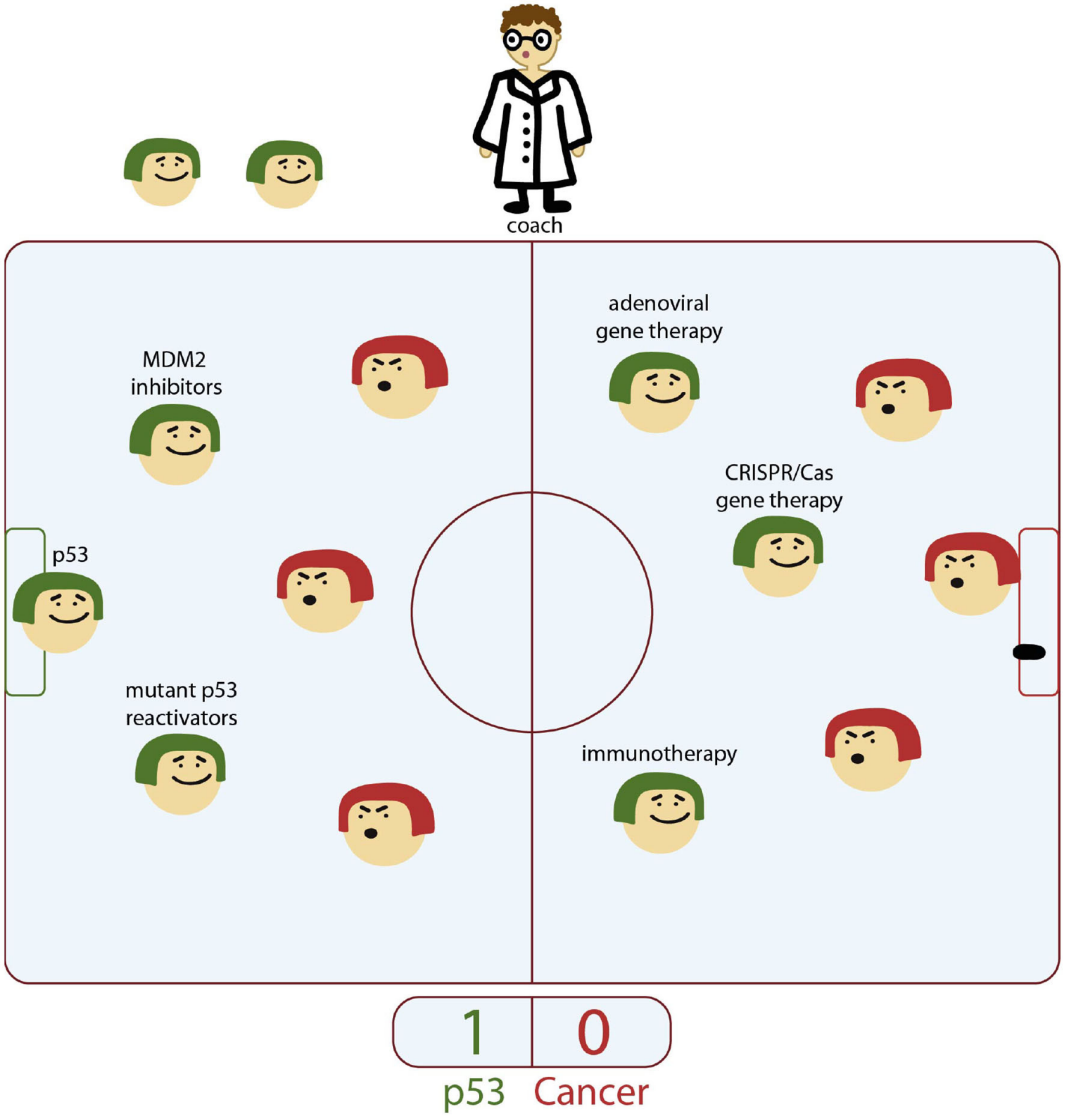
Key players in the p53 team. P53 is the genome “gatekeeper.” MDM2 inhibitors, mutant p53 re-activators are the players of defensive line, whereas adenoviral gene therapy, gene editing tools, and immunotherapy are part of the offensive line in p53 team. New and yet unknown powerful players are expected to enter the game at the forefront of cancer treatment and score a success under the researcher coaching.

The general approaches employed to destroy the p53-mutant tumor cells are implemented either *via* restoration of its WT oncosuppressor properties, or focus on tumor elimination by manipulating key components of the immune system. In this review we discuss the current and emerging therapeutic strategies against mutant-p53-driven cancers based on small molecule re-activators, gene editing technologies (introduction of WT gene or CRISPR/Cas mediated corrections) and T cell immunotherapy (Figure 1).

## Defensive Strategy: Small Molecule Re-Activators

MDM2 is mostly known for its oncogenic properties, though its role beyond cancer, notably inflammation, has received increasing attention in recent years (8–10). Numerous synthetic modulators that activate WT p53 by MDM2-dependent, e.g., Nutlin-3a (11–13), and MDM2-independent mechanisms (14–16) have been reported. However, Nutlins and similar inhibitors of MDM2 often demonstrated side effects in clinical trials, such as off-target issues and dose-limiting hematological toxicities, e.g., thrombocytopenia and neutropenia.

Chemoresistant MDM2 mutations were also reported to evolve, although there is evidence that this may be addressed by combination therapies using stapled-peptide MDM2 antagonists. Such mutations occur in *N*-terminal p53-binding domain, zinc finger and RING domains. “Stapled” peptide inhibitor (PM2) has been reported, which has a covalent hydrocarbon linkage bridging the adjacent turns of an alpha helical peptide for improved stability (17). The peptide recapitulated key p53 signature residues and targeted the *N*-terminal domain of MDM2. The structural mimicry and extended spatial contacts with the protein allowed PM2 to retain binding (K_D_ = 117 nM) to mutant forms of MDM2 resistant to Nutlins.

**Figure 2:**
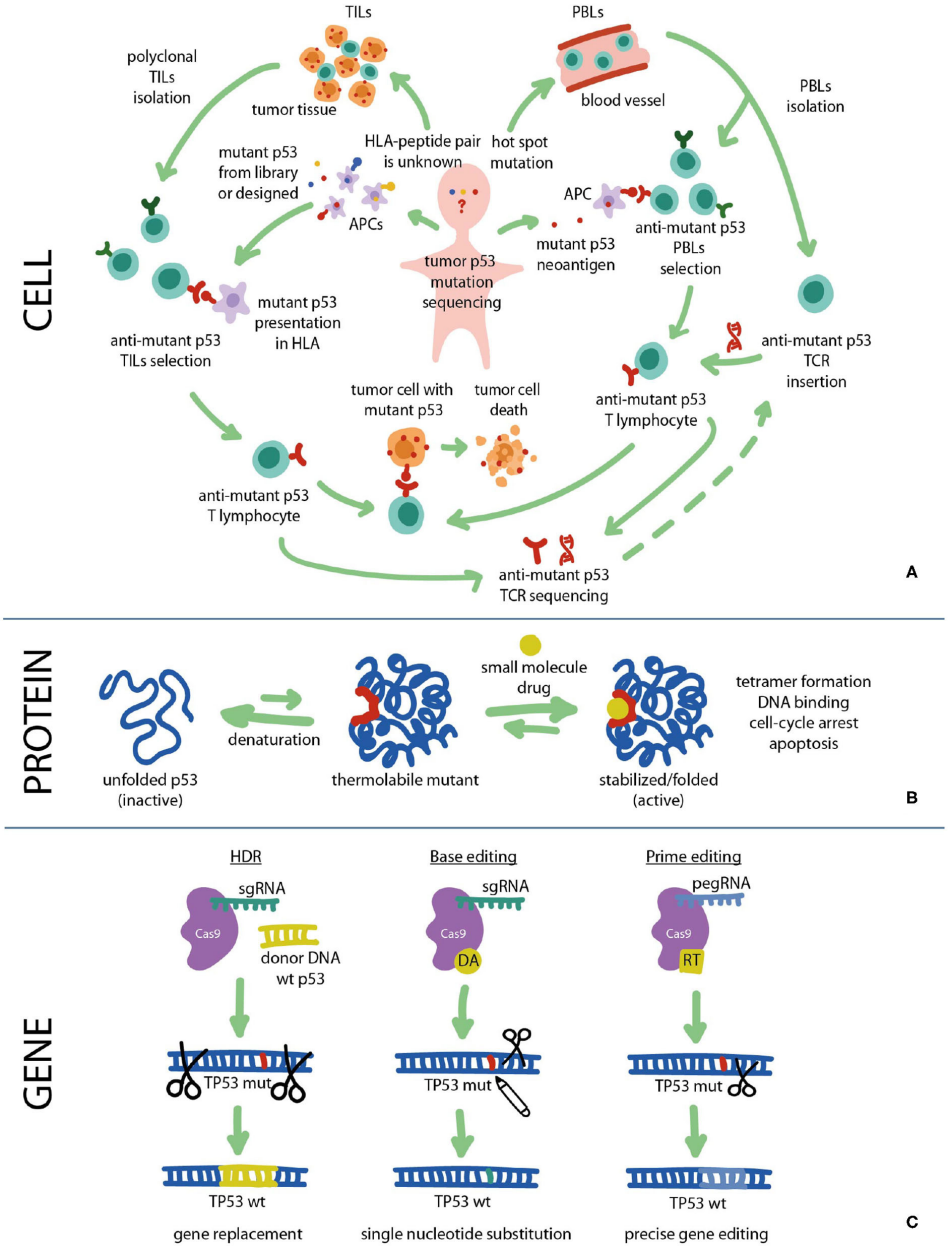
Fighting cancer via p53 pathway can be implemented at all levels: cell, protein, and gene. **(A)** Cancer cells carrying mutant p53 can be targeted with immunotherapy using mutant p53-specific TILs or TCR-T cells. **(B)** At the protein level the DNA-binding and transcriptional functions of mutant p53 can be restored using small molecule re-activators that stabilize the protein in its active biological conformation. **(C)** At the gene level *TP53* mutations can be repaired using CRISPR/Cas9 gene editing approaches such as HDR, Base editing and Prime editing. HDR, homology directed repair; HLA, human leukocyte antigen; PBL, peripheral blood lymphocyte; RT, reverse transcriptase; TCR, T cell receptor; TIL, tumor-infiltrating lymphocyte.

Targeting tumors with mutant *TP53*, both somatic and germline, presents a challenging yet potentially highly rewarding approach as such mutations are the main driver of various types of cancer (18). The equilibrium between the properly folded and misfolded states of p53 can be affected by compounds that interact with mutant p53 and reinstate its native fold and function (Figure 2B). A number of small molecules have been developed to target and stabilize specific mutant forms of p53 and restore WT resembling transcriptional activity, thereby leading to cell cycle arrest or apoptosis of mutant tumor cells. While many tumor suppressor genes are predominantly inactivated in cancer through deletion, truncating mutations or epigenetic mechanisms, the majority of p53 cancer mutations are missense mutations which lead to the expression of functionally altered full-length mutant p53 proteins with single amino acid substitutions. Approximately one third of oncogenic p53 mutants are conformationally unstable due to specific “hot spot” residues that are mutated at a disproportionately high frequency, most of which reside in the structured p53 DNA-binding region (19). The nine most frequent mutations (R175H, R248Q, R273H, R248W, R273C, R282W, G245S, R249S, Y220C), the majority of which are DNA contact mutants, account for about 30% of all p53 cancer driving mutations.

Such “contact” mutants not only lose their transcriptional activity due to impaired DNA binding, but also exhibit dominant-negative (DN) effects on the remaining WT p53 allele in addition to the homologous tumor-suppressors p63 and p73 (20). Mutant p53 proteins can form heterotetramers with WT p53, hampering the function of the latter in tumor suppression (21). The primary outcome of *TP53* mutations leading to loss of WT p53 functions is the abrogation of its intrinsic tumor suppressive responses such as senescence and apoptosis, while gain-of-function mutant p53 proteins enhance tumor progression, metastatic potential, and drug resistance, greatly contributing to the malignant cellular phenotype (22–24).

Most p53 mutants lose their transcriptional activity and tumor suppressive function, although approximately a third of p53 mutants are temperature sensitive and display sequence-specific transcriptional activity at sub-physiological temperatures (25, 26). Interestingly, introduction of rationally designed second-site suppressor mutations was shown to stabilize the structure of the p53 DBD and reactivate transcription, providing access to valuable WT like variants for screening and drug discovery (27, 28). At the same time, this suggests that stabilization of such “conformational” mutants may provide an opportunity to reinstate their WT function through the use of modulators of their thermal stability. There is currently enormous interest in the identification of natural or synthetic substances (small molecules, peptides, etc.) that can stabilize mutant p53 in its active biological conformation and restore DNA-binding and transcriptional activity (29).

PRIMA-1 and its methyl analog APR-246 (PRIMA-1^MET^) are promising small molecules that can restore activity of mutant p53 by interacting with the DNA binding domain, promoting proper folding/function (29). This leads to enhanced expression of pro-apoptotic genes *Puma, Noxa*, and *Bax* in p53 mutant cells in addition to activation of cell-cycle genes and PARP cleavage independent of p53 mutation status, as observed in multiple studies that involved various types of cancer such as breast, thyroid, myeloma (30).

Both PRIMA and APR-246 are pro-drugs that are intracellularly converted to the reactive methylene quinuclidinone (MQ), which covalently binds to surface-exposed cysteine residues of mutant p53 as well as WT p53. At the same time, experiments with recombinantly expressed and intracellular p53 proteins have shown that unfolded mutant p53 was modified by PRIMA-1 more efficiently than the correctly folded WT protein (31). MQ may also exert its anticancer effect *via* an alternative p53-independent mechanism of action based on glutathione (GSH) depletion, leading to upregulation of reactive oxygen species (ROS) levels and modulation of the intracellular redox state (32). Currently, APR-246 in combination with azacitidine has reached Phase III clinical trial for the treatment of *TP53* mutant myelodysplastic syndromes (MDS) (NCT03745716) and Phase II for *TP53* mutant myeloid neoplasms (NCT03072043, NCT03588078).

Bauer et al. (33) identified a range of 2-sulfonylpyrimidines as mild arylating agents of surface cysteines in both WT p53 and mutant p53 core domains. Cysteine arylation upon treatment with lead molecule PK11007 stabilized the mutant p53 core domain *in vitro* by up to 3°C in differential scanning fluorimetry experiments. In cells, it induced concentration-dependent upregulation of several p53 target genes (*p21, PUMA*) in cancer cell lines, although p53-independent cytotoxicity was also observed in p53-null and WT p53 cell lines. Interestingly, PK11007 also induced strong GSH depletion and ROS upregulation in cells, reminiscent of the cellular profile and suggested mode of action of MQ and its derivatives. Altogether, these studies highlighted the important effect of cellular redox modulation and a potential general strategy for the development of covalent anticancer agents targeting mutant p53 and redox pathways synergistically, although the propensity for off-target redoxive cell damage by such agents is high.

The Y220C mutation is the ninth most frequent p53 missense mutant overall and is associated with over 100,000 new cancer cases per year worldwide, predominantly breast and ovarian cancer (18, 34). Behind the most common “contact” mutations (*vide infra*), it is by far the most frequent “conformational” p53 cancer mutation. This large-to-small residue mutation creates an extended cavity on the protein surface that destabilizes the DBD by ~4 kcal/mol (35), causing denaturation and aggregation. The hydrophobic and “druggable” nature of the Y220C pocket offers a fruitful opportunity for targeting using small-molecule stabilizers. Critically, the mutation-induced crevice is distant from the p53 surfaces involved in DNA recognition or protein-protein interactions, allowing for the development of targeted chemical agents that stabilize the DBD without interfering with binding of its natural substrates.

In recent years, fragment-based and *in silico* screening methods have led to the identification of several potent lead compound families that bind the Y220C pocket. A range of carbazole derivatives displaying low micromolar affinity increased the melting temperature of p53-Y220C and slowed its rate of aggregation *in vitro*. PK9328 (*K*_D_ = 2 μM) induced cell viability reduction of several Y220C cancer cell lines, although some toxicity was also observed in other cell lines not carrying this mutation, possibly suggesting off-target effects (36). Pyrazole derivative PK7088 rescued the folding of p53-Y220C and restored transactivation and downstream upregulation of *p21* and *Noxa* expression, correlating with cell cycle arrest and apoptosis (37).

Recently, our group reported several potent iodophenol lead molecules displaying low micromolar binding affinity *in vitro*, thermal stabilization of up to 2.2°C and selective pro-apoptotic activity in a panel of Y220C cancer cells. Structure-activity studies culminated in aminobenzothiazole derivatives MB710 and MB725, which demonstrated *in vitro K*_D_ up to 4 μM for p53-Y220C by isothermal titration calorimetry (38). MB725 also showed potent and selective viability reduction of several p53-Y220C cancer cell lines such as BXPC-3 (pancreatic adenocarcinoma), HUH-7 (hepatocellular carcinoma), NUGC3 (gastric adenocarcinoma), while maintaining comparatively low toxicity in WT p53 WI38 (normal fibroblasts), and NUGC4 (gastric adenocarcinoma) in the same concentration range. Importantly, the correlation between *in vitro* thermal stabilization and selective viability reduction in Y220C cell lines represents an important milestone toward first-in-class anticancer drugs that rescue p53-Y220C function. This provides a compelling rationale for future lead optimization efforts toward potent, non-toxic targeted agents for reactivating the Y220C mutant in anticancer therapy.

ZMC-1 (zinc metallochaperone-1) is a thiosemicarbazone-based small molecule that rescues the WT protein folding and transcriptional activity of p53-R175H mutant by buffering the intracellular Zn^2+^ levels (39). The underlying rationale is that zinc is required for the correct folding of WT p53 protein and mutations that impair zinc binding strength can hamper protein stability and conformation, leading to impaired sequence-specific DNA binding to p53 response elements (3, 40). ZMC-1 restored site-specific DNA binding and upregulation of p53 target genes (*p21, Puma, Mdm2*) (41), and inhibited mouse xenograft tumor growth with high allele-specificity for the p53-R175H (p53-R172 in mice) mutant. While zinc buffering alone was insufficient to induce apoptosis (41), ZMC-1 also activated p53 by induction of ROS through its ability to chelate other metal ions (Fe^2+^, Fe^3+^, Cu^2+^) (42). The 3rd-generation thiosemicarbazone COTI-2 functions similarly through both p53-mediated pathways and p53-independent redox homeostatic mechanisms (43) and has entered a Phase II clinical trial (NCT02433626), although it is of note that thiosemicarbazone cancer drug candidates have known nonspecific cytotoxicity and effects on iron metabolic pathways (44).

## Offensive Strategy: Gene Therapy and Immunotherapy

### Adenoviral Gene Therapy

Gene therapy is a promising therapeutic option and some practical examples have already been studied and successfully applied to re-establish WT p53 expression and activity in cancer cells. Gene therapy involves the replacement or addition of a correct copy of the abnormal gene with a view to restore the genetic information, thus reinstating the WT phenotype.

Currently, gene therapy approaches are based on the combination of genetic material with suitable delivery systems that are often limited by the requirement for efficient nuclear delivery and gene expression. Several primary delivery systems for *TP53* gene-based therapeutics have been developed using various viral vectors, including adenoviral, retroviral, vaccine-derived vectors and non-viral ones based on liposomes, polymeric, and gold nanoparticles that allow overcoming systemic delivery hurdles (45). Currently, adenoviral vectors demonstrate minimum side effects among viral vectors used for *TP53* gene therapy.

Up until now, several clinical studies using viral vectors for the delivery of p53 have been conducted for experimental medicines, such as Advexin and Gendicine. Advexin (Introgen Therapeutics Inc., TX, USA) is an adenoviral-based experimental therapeutic that provided delivery of WT p53 to cancer cells and demonstrated anticancer activity following amended expression of p53 (46). Gendicine, based on recombinant human p53 adenovirus (Shenzhen SiBiono GeneTech Co. Ltd., China), was approved in 2003 by the China Food and Drug Administration (CFDA) as a first-in-class gene therapy product to treat head and neck cancer, and entered the commercial market in 2004 (47).

Novel adenoviral vectors for cancer gene therapy targeting the p53 pathway were developed to improve the transgene expression levels. Two adenoviral vectors were reported that differ only in the promoter site: the constitutive CMV promoter and the p53-responsive PG promoter where a p53-responsive element is inserted in the viral vector (48). The p53 expression was found to be substantially higher in PCa cells after transduction with AdPGp53 compared to AdCMVp53, and DU145 cells were particularly susceptible to the AdPGp53 tumor suppressor properties.

However, the application of viral vectors can induce high immunogenicity and enhance pre-existing immunity, which limits their clinical use and requires development of new systems with equal efficiency but better safety profiles. Non-viral vectors could present significant advantages when compared with viral ones due to their safety and low cost; nevertheless, viral vectors currently dominate gene therapy clinical trials because of their relatively high delivery efficiency. Thus, viral vectors for the delivery of WT *TP53* gene are seen as strong players in the p53 team, however, introduction of other powerful players would increase the firepower of the offensive line.

### CRISPR/Cas Gene Editing

There are numerous molecular tools for programmable genome editing at a clinical level, including zinc-finger nucleases (ZFNs) (49, 50), transcription activator-like effector nucleases (TALENs) (51, 52), clustered regularly interspaced short palindromic repeats (CRISPR)/CRISPR-associated (Cas) (53). CRISPR/Cas is widely seen as a revolutionary technology for biomedical research with immense clinical opportunities for treating cancer and genetic disorders.

In 2016 the laboratory of David Liu at Harvard University developed an advanced version of CRISPR/Cas enzymes, called Base Editors (BEs), which can mediate specific point mutations in genomic DNA and the resulting amino acid sequence of a target protein (54, 55). BEs constitute enzymatically inactive Cas9 nickase (nCas9) fused to either cytidine deaminase (cytidine BE) or adenosine deaminase (adenosine BE) that result in cytosine-to-thymine or adenine-to-guanine conversion in DNA. In human cells BEs function with high efficiency (15–75%) and low indel rates (<0.1%) compared to classical CRISPR/Cas9 technique based on homology directed repair (HDR). BEs could significantly advance treatment of mutation-associated cancer and genetic diseases by specifically correcting pathogenic mutations in the target gene.

In 2019 the same laboratory reported new gene editing tool, Prime Editors (PEs), based on even more advanced CRISPR/Cas9 “search-and-replace” technology (56). Here, the desired genetic information is directly introduced using nCas9 fused to reverse transcriptase that is directed by prime editing guide RNA (pegRNA) specifying the target DNA sequence and encoding the genetic edits. PEs expand the list of available genome editing tools and together with BEs they can potentially correct ~89% of all known pathogenic human genetic variants.

Several clinical trials are in progress to apply CRISPR/Cas9 for the treatment of patients with mutation-associated disorders, such as β-thalassemia (NCT03655678, NCT03728322) and sickle cell disease (NCT03745287) whereby genetic manipulations with blood cells are carried out *ex vivo* and then gene-corrected cells are infused back to the patient. A particularly remarkable example is Leber congenital amaurosis 10 (NCT03872479), for which CRISPR-based investigational therapy is administered *in vivo* via subretinal injection.

Oncogenic or disease-causing mutations represent the primary targets for gene editing therapies. The highest mutation rate of *TP53* among other genes makes it a highly desirable target for gene editing tools, e.g., to reverse missense mutation back to the WT state. Chira et al. (57) proposed a CRISPR-based delivery system of a functional *TP53* gene. According to the authors, the entire mutated *TP53* locus could be deleted and then replaced with a functional copy by homologous recombination. In principle, this might be feasible because the CRISPR/Cas9 system is capable of making such large insertions (58). As a result, the WT phenotype of *TP53* could be recovered by replacing the perturbed gene with its functional copy leading to normal p53 expression and tumor regression.

CRIPSR/Cas9 gene editing, including Base Editing, Prime Editing and upcoming technologies have set a high expectations bar for future clinical applications (Figure 2C). BEs, PEs and similar approaches that allow introduction of precise genetic corrections into a target locus without deleting the whole gene could potentially be used to correct *TP53* missense mutations as a prospective anticancer therapy (59). Given the rapid advancement of CRISPR/Cas9 technologies and their inevitable introduction to clinical practice, both *ex vivo* and *in vivo* target gene modifications in a wide range of cancers, including solid tumors, does not seem to be a distant future anymore.

However, efficient intracellular delivery remains one of the main barriers on the path for wider clinical application of CRISPR/Cas9 technology, including for the purposes of therapeutic editing of *TP53* gene. There are three primary strategies for intracellular delivery of CRISPR/Cas9 components: viral vectors, lipid nanoparticles and Cas9-sgRNA complexes. Among these the viral gene delivery strategy seems to be the closest to clinical practice because it has been used in classical gene therapy for decades (60). CRISPR/Cas9-induced double strand breaks (DSBs) of the genomic DNA can result in cell cycle arrest or cell death through p53 pathway that induces DNA damage response and activates expression of downstream effector proteins, e.g., cell cycle inhibitor p21^CIP1/WAF1^. Functioning of the cellular DNA repair mechanisms that get activated upon DSB, which is often an integral initial step of the gene-editing mechanism, explains one of the reasons for low efficiency of the classical CRISPR/Cas9 system (61, 62).

The rapid development of CRISPR/Cas9-based technologies for therapeutic gene editing of the *TP53*-associated pathologies is expected to enhance precision, enable improved correction of point mutations, provide better delivery, reduce side effects and facilitate wider clinical applications.

### Immunotherapy

*TP53* mutations as part of the overall tumor mutational burden (TMB) can be considered an important factor in predicting response to immunotherapy. *TP53* missense mutation-associated p53 nuclear accumulation results in a higher local density of tumor-infiltrating lymphocytes (TILs) within the primary tumor (63). The p53 protein can regulate the immune landscape by modulating inflammation, senescence and immunity in the surrounding tumor microenvironment (TME), including tumor stroma, extracellular matrix (ECM) and associated immune cells infiltrate (64). Mutation in p53 can lead to enhanced neo-angiogenesis and ECM remodeling, disruption of innate tumor immunity, genotoxic stress response of the Toll-like receptor (TLR) pathway, favor pro-tumor macrophage signature and alter cell-mediated immunity in cancer (65).

Some pathways leading to T cell exhaustion are upregulated in such tumors, therefore making them a good target for immunotherapeutic treatment based on genetically modified T cells, e.g., T cell receptor (TCR)-T cells or chimeric antigen receptor (CAR)-T cells (66).

Tumor cells elicit immunogenic responses due to “hot spot” mutant p53 epitopes (neoantigens) produced *via* proteasomal degradation of intracellular protein and presented by major histocompatibility complex (MHC) (Figure 2A). Initial studies showed that tumors with mutated *TP53* could be recognized by peripheral blood lymphocytes (PBLs) upon *in vitro* stimulation and *in vivo* immunization (67–69). Cancer vaccines based on primed autologous dendritic cells (DCs) reactive to neoepitopes lead to enhanced antitumor T cell responses in ovarian cancer patients and were associated with better survival prognosis (70).

Tumor-specific adoptive cell therapy (ACT) using antigen-experienced T cells, e.g., patient's own autologous TILs, is a novel approach for targeting p53 mutant cancers. In this approach a HLA/neoantigen complex is recognized by T cell receptors (TCRs) of cytotoxic T cells that effect tumor lysis. Particularly interesting are genetically-engineered T cell receptor (TCR)-T cells with known HLA/neoantigen combination generated by transduction or transposition of specific TCRs into autologous or allogeneic T cells (71). Limitations of this method include differentiation status and proliferative potential of TILs/TCR-Ts, and most importantly potential loss of HLA on tumor cells that would restrict the efficiency of T cell-mediated cytotoxicity.

Deniger et al. (72) prospectively evaluated intratumoral T cell responses to autologous somatic mutant p53 neoantigens expressed by human metastatic ovarian cancers. T cells with specificity to mutated neoantigens found in high frequencies in TILs were expanded from resected metastases and then co-cultured with autologous antigen-presenting cells (APCs) expressing mutated p53 epitopes (Y220C and G245S). Immunogenicity of T cell response was confirmed by upregulation of 4-1BB or secretion of IFNg.

Lo et al. (73) screened TILs for recognition of mutated neoantigens in metastatic colorectal cancer patients and observed T cell mediated recognition of immunogenic p53-R175H mutant. Several TCRs were also identified that could be transduced into allogeneic PBLs for ACT application as an off-the-shelf TCR-T cell product targeting cancer cell lines with a wide range of *TP53* mutations.

Malekzadeh et al. (74) developed a *TP53*-specific screening assay to evaluate T cell responses to “hot spot” mutant p53 neoantigens introduced to autologous APCs intracellularly (tandem minigenes) or extracellularly (pulsed peptides). TCRs from CD4+ and CD8+ T cells reactive to mutant p53 neoantigens were identified in lung cancer patients and then TCR-T cells were engineered that recognize the same HLA/neoantigen complex. In follow-up experiments they isolated PBLs from patients with mutant p53 (R175H, Y220C, R248W) tumors by sorting antigen-experienced CD4+ and CD8+ T cells (75). The T cells were then stimulated with p53 neoantigens (naturally occurring processed and presented peptides) *in vitro* to confirm the recognition and specificity of the immune response.

Future studies will reveal detailed mechanisms of the complex regulatory interplay between the tumor *TP53* status and the immune landscape, including p53-mediated innate anti-tumor response and presentation of mutant p53 neoantigens for eliciting immune recognition by T cell receptors.

## Conclusion

The set of available molecular tools arming scientists to battle somatic mutation-associated tumors and hereditary diseases has expanded significantly in recent years. Traditional approaches such as rational structure- and fragment-based drug discovery targeting protein interfaces have been successfully complemented with innovative gene- and cell-based technologies. Adenoviral gene therapy and CRISPR/Cas gene editing are advancing in clinical trials for the treatment of mutation-linked diseases, and the expansion of their applications for therapeutic targeting of *TP53* mutations inevitably also approaches. Immunotherapy based on genetically engineered T cells (either autologous or allogeneic) complement cancer treatment by providing unique specificity and efficiency. Therefore, the key players in the mutant p53 team—small molecules, adenoviruses, CRISPR/Cas gene editing enzymes, T cell-based therapies and combinations thereof—broaden the therapeutic scope and provide enormous clinical potential for targeting p53 mutant tumors at all levels (gene, protein and cell). We believe that these approaches have truly encouraging opportunities for clinical applications and that major advancements based on them are approaching in the near future. Together they will fuel challenging, but highly rewarding new developments in the field of mutant p53 cancer therapy.

## Author Contributions

VC, JS, and MB contributed to section about small molecules. RM and RK contributed to section about gene editing. AV and EZ contributed to section about immunotherapy. EZ and RM prepared the figures. VC, AR, MB, and EB contributed to introduction and conclusion. VC, MB, and EB conceived the idea and coordinated the writing. All authors contributed to the article and approved the submitted version.

## Conflict of Interest

The authors declare that the research was conducted in the absence of any commercial or financial relationships that could be construed as a potential conflict of interest.

## Abbreviations

ACT: adoptive cell therapy
APC: antigen-presenting cells
BE: base editor
CAR: chimeric antigen receptor
CFDA: China Food and Drug Administration
CRISPR/Cas: clustered regularly interspaced short palindromic repeats/CRISPR-associated
DBD: DNA-binding domain
DC: dendritic cell
DSB: double strand break
ECM: extracellular matrix
HDR: homology directed repair
MDM2: murine double minute 2
MDS: mutant myelodysplastic syndrome
MHC: major histocompatibility complex
MQ: methylene quinuclidinone
nCas9: Cas9 nickase
PBLs: peripheral blood lymphocytes
PE: prime editor
pegRNA: prime editing guide RNA
ROS: reactive oxygen species
TALEN: transcription activator-like effector nuclease
TCR: T cell receptor
TILs: tumor-infiltrating lymphocytes
TMB: tumor mutational burden
TME: tumor microenvironment
TLR: Toll-like receptor
WT: wild-type
ZFN: zing-finger nuclease
ZMC: zinc metallochaperone.
